# Metal Hyperaccumulation Armors Plants against Disease

**DOI:** 10.1371/journal.ppat.1001093

**Published:** 2010-09-09

**Authors:** Helen Fones, Calum A. R. Davis, Arantza Rico, Fang Fang, J. Andrew C. Smith, Gail M. Preston

**Affiliations:** Department of Plant Sciences, University of Oxford, Oxford, United Kingdom; The University of North Carolina at Chapel Hill, United States of America

## Abstract

Metal hyperaccumulation, in which plants store exceptional concentrations of metals in their shoots, is an unusual trait whose evolutionary and ecological significance has prompted extensive debate. Hyperaccumulator plants are usually found on metalliferous soils, and it has been proposed that hyperaccumulation provides a defense against herbivores and pathogens, an idea termed the ‘elemental defense’ hypothesis. We have investigated this hypothesis using the crucifer *Thlaspi caerulescens*, a hyperaccumulator of zinc, nickel, and cadmium, and the bacterial pathogen *Pseudomonas syringae* pv. maculicola (*Psm*). Using leaf inoculation assays, we have shown that hyperaccumulation of any of the three metals inhibits growth of *Psm in planta*. Metal concentrations in the bulk leaf and in the apoplast, through which the pathogen invades the leaf, were shown to be sufficient to account for the defensive effect by comparison with *in vitro* dose–response curves. Further, mutants of *Psm* with increased and decreased zinc tolerance created by transposon insertion had either enhanced or reduced ability, respectively, to grow in high-zinc plants, indicating that the metal affects the pathogen directly. Finally, we have shown that bacteria naturally colonizing *T. caerulescens* leaves at the site of a former lead–zinc mine have high zinc tolerance compared with bacteria isolated from non-accumulating plants, suggesting local adaptation to high metal. These results demonstrate that the disease resistance observed in metal-exposed *T. caerulescens* can be attributed to a direct effect of metal hyperaccumulation, which may thus be functionally analogous to the resistance conferred by antimicrobial metabolites in non-accumulating plants.

## Author Summary

Soils rich in heavy metals support communities of distinctive metal-tolerant plants, a number of which exhibit a remarkable trait known as metal hyperaccumulation. These plants accumulate exceptionally high concentrations of metallic elements in their leaves, but whether this trait confers any adaptive advantage is controversial. In this study, we test the hypothesis that metal hyperaccumulation provides protection against disease. We demonstrate that *Thlaspi caerulescens* becomes resistant to bacterial leaf spot caused by *Pseudomonas syringae* pv. maculicola (*Psm*) when it accumulates zinc, nickel, or cadmium, and show that the metal concentrations in these plants are sufficient to account for their observed disease resistance. We also show that there is a close correlation between the zinc tolerance of different strains of *Psm* and their ability to colonize *T. caerulescens* leaves with high zinc content. In a field study, bacteria isolated from the leaves of *T. caerulescens* plants growing on the site of a former lead–zinc mine were found to possess a higher degree of metal tolerance than bacteria isolated from crop plants. Our findings show that metal hyperaccumulation in plants can act as a mechanism to prevent attack by pathogenic microorganisms, but that metal tolerant pathogens can overcome this defense.

## Introduction

Metal hyperaccumulation is described as the accumulation of exceptionally high concentrations of metallic elements in the aerial parts of a plant [1], [2]. The phenomenon has evolved in around 450 plant species, distributed across several families [2], [3], [4], and most hyperaccumulator species are endemic to metalliferous soils, either natural or anthropogenic in origin [5]. This unusual characteristic has attracted considerable interest, and a number of hypotheses have been proposed to explain the evolution of the hyperaccumulation phenotype [6], [7]. The possibility that accumulated metal provides a defense against herbivores or pathogens, termed the ‘elemental defense hypothesis’, has received most attention and support [7], [8], [9].

A number of studies have reported findings consistent with a defensive effect of hyperaccumulated metals against herbivores [10], [11], [12], but the interpretation of these results remains controversial, as other studies have failed to support the defense hypothesis. For instance, Noret et al. [13], [14] were unable to find a defensive effect attributable to metal hyperaccumulation in the field, despite that reported in laboratory trials. There is also some evidence that the importance of metal-based defense is dependent upon the mode of herbivory [15]. In the case of defense against pathogens, considerably fewer tests have been carried out [e.g. [16]] and only one study [17] has explicitly tested the defense hypothesis with regard to bacterial pathogens. So far, although some evidence has been provided that plants exposed to high metal concentrations have reduced susceptibility to various pathogens, no study has demonstrated that the metal itself was directly responsible for this effect. It is therefore not possible, at present, to state that metal hyperaccumulation trait evolved as an antimicrobial defense.

Here, we test the possibility of a direct elemental defense against bacterial pathogens in the crucifer *Thlaspi caerulescens*, a hyperaccumulator plant characteristically associated with metalliferous soils [18]. In its natural habitats, this species can hyperaccumulate three different metals: zinc, nickel, and cadmium [19], [20], and it has been widely used in studies of the hyperaccumulation trait [21], [22], [23], [24], [25].

We have tested the elemental defense hypothesis *via* a three-pronged approach by means of studies *in vitro*, *in planta*, and in the field. First, we have monitored the growth of *Pseudomonas syringae* pv. maculicola M4, a model pathogen of *Arabidopsis thaliana* [26], in *T. caerulescens* plants treated with different metal concentrations. This pathogen grows in the apoplastic spaces between plant cells, so we have investigated metal concentrations in the apoplastic phase specifically to determine whether they are likely to be inhibitory to pathogen growth. A number of studies attempting to determine the location of hyperaccumulated metals within the leaf have found the majority to be accumulated in the epidermal vacuoles [27], [28], [29], but apoplastic metal concentrations have not been examined in previous studies. Further, we have assessed the growth of *Psm* mutants with altered zinc sensitivity in *T. caerulescens* plants grown on different zinc regimes. This has allowed us to test whether zinc tolerance is important for bacterial growth *in planta*, as expected if metals are directly involved in plant defense. Finally, we have tested the zinc tolerance of endophytic bacteria found in the leaves of *T. caerulescens* growing on a metal-rich soil at the site of a former lead–zinc mine to determine whether there is evidence that this form of defense may be effective under natural field conditions.

## Results

### *Pseudomonas syringae* pv. maculicola M4 is a pathogen of *Thlaspi caerulescens*

**Figure 1:**
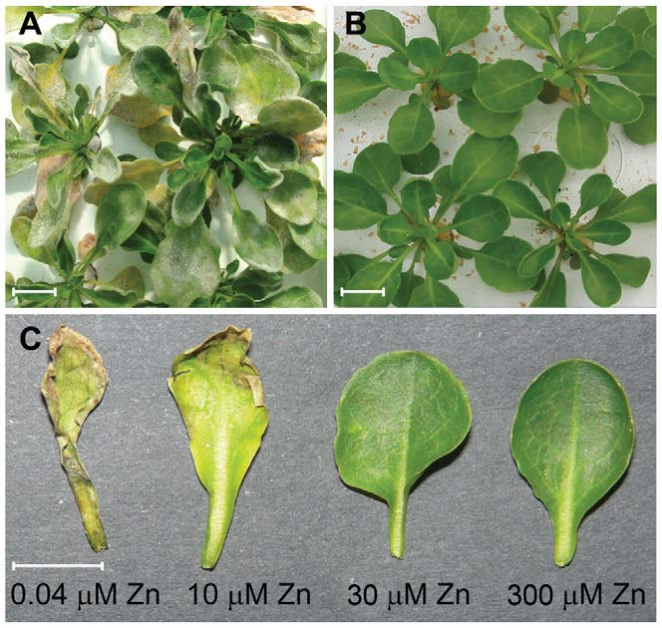
High zinc concentrations suppress disease symptoms in *Thlaspi caerulescens*. **A**. *T. caerulescens* plants growing on 10 µM zinc during an outbreak of mildew (*Erysiphe* sp.) in the glasshouse. **B**. *T. caerulescens* plants growing on 300 µM zinc during the same outbreak of mildew in the glasshouse. **C**. *T. caerulescens* plants were grown for 10 weeks on nutrient solution containing 0.04, 10, 30, or 300 µM ZnSO_4_. Leaves were infiltrated with *P. syringae* pv. maculicola M4 suspended in 10 mM MgCl_2_ at 10^8^ cfu/ml and photographed 96 hours after inoculation. Scale bars represent 10 mm.

When *Thlaspi caerulescens* plants were grown in the glasshouse, it was observed that occasional outbreaks of powdery mildew affected only those plants growing on low metal treatments (Figure 1A and B). This observation prompted us to consider the importance of metals in defending *T. caerulescens* against pathogens. As powdery mildews are obligate biotrophs and therefore unculturable, we sought a more tractable model system for further study of this phenomenon. Bacterial plant pathogens are suitable for such investigations because they are easily cultured *in vitro* and are relatively straightforward subjects for genetic manipulations such as mutagenesis.

**Figure 2:**
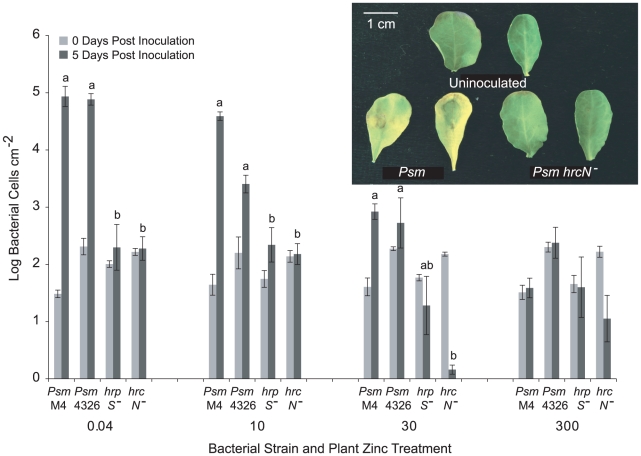
T3SS mutants of *Pseudomonas syringae* pv. maculicola are unable to colonise *Thlaspi caerulescens*. *P. syringae* pv. maculicola M4 (*Psm* M4), *P. syringae* pv. maculicola ES4326 (*Psm* 4326) and two T3SS mutants of *Psm* (*hrpS^−^* and *hrcN^−^*) were inoculated into fully expanded leaves of *T. caerulescens* at 10^6^ cfu/ml. Three samples were taken for each strain, zinc treatment and time point, each consisting of three leaf discs pooled together. Values are means±SE (*n* = 3). The mean growth of the four strains at each zinc treatment over 5 days post-inoculation was compared in ANOVAs; growth of the four strains was found to differ significantly in all treatments except in the 300 µM zinc treatment, where no strain was able to grow (*P*<0.0005 for 0.04 and 10 µM Zn; *P* = 0.001 for 30 µM Zn; *P* = 0.169 for 300 µM Zn). Within each zinc treatment, Bonferroni simultaneous comparisons were used to determine which means differed significantly at 5 days post-inoculation, and these are marked with different letters. The inset shows symptoms observed in leaves from *T. caerulescens* plants grown on 0.04 µM zinc 72 hours after inoculation with wild-type *Psm* ES4326 and the *Psm hrcN^−^* mutant at 10^6^ cfu/ml compared with uninoculated control leaves.

Of sixteen plant pathogenic bacteria tested, eight were found to cause necrotic symptoms in *T. caerulescens* within 2 to 4 days after inoculation [see Supporting Information Table S1]. *Pseudomonas syringae* pv. maculicola M4 (*Psm*), a rifampicin-resistant derivative of *Psm* 4326 [26], caused necrosis more rapidly and to a greater extent than any other tested strain. To confirm that *Psm* is pathogenic on *T. caerulescens*, we assessed the ability of *Psm*, *Psm* ES4326 (a streptomycin resistant derivative of *Psm* 4326) [30] and two type III secretion system (T3SS) mutants of ES4326 (gift of K. Schreiber and D. Desveaux) to grow in *T. caerulescens*. Over 5 days post-inoculation, *Psm* M4 and *Psm* ES4326 both multiplied two to three logs in the leaves of *T. caerulescens* plants grown on minimal (0.04 µM) zinc. However, neither T3SS mutant was able to grow in *T. caerulescens* at any of the zinc concentrations tested (Figure 2). Both T3SS mutants grew similarly to wild-type bacteria *in vitro* (Figure S1). This confirms that *Psm* is pathogenic towards *T. caerulescens* and shows that the ability of *Psm* to grow in *T. caerulescens* is T3SS-dependent.

### Hyperaccumulation of zinc, nickel, or cadmium inhibits the growth of *Pseudomonas syringae* pv. maculicola M4 in *Thlaspi caerulescens*

On inoculation of *Psm* into *T. caerulescens* plants treated with progressively higher concentrations of zinc, both symptom development and *Psm* growth were significantly reduced as the concentration of the zinc treatment increased (Figure 1C and D; Figure 2).

**Figure 3:**
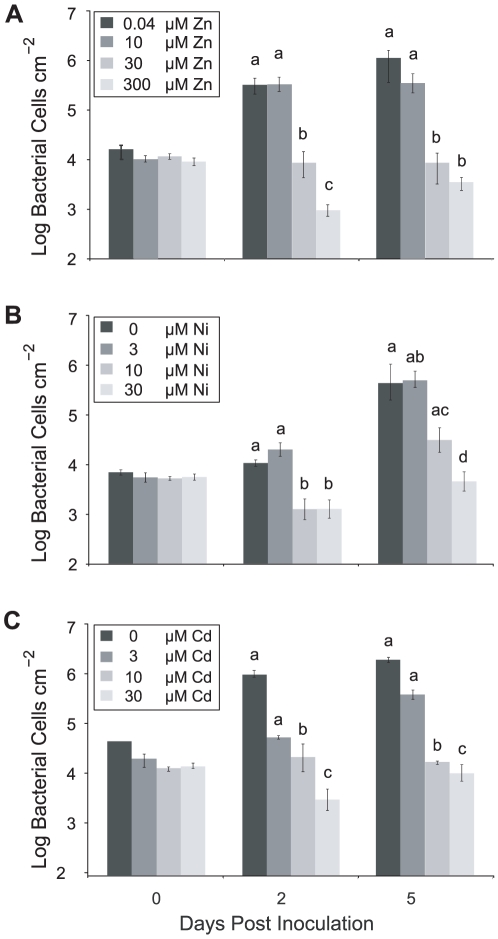
High metal concentrations inhibit bacterial growth in *Thlaspi caerulescens*. *T. caerulescens* plants were treated with a range of zinc (**A**), nickel (**B**), or cadmium (**C**) concentrations. Nine leaves of each of six plants were infiltrated with *P. syringae* pv. maculicola M4 suspended in 10 mM MgCl_2_ at 10^6^ cfu/ml and leaves sampled at 0, 2 and 5 days after inoculation. Six samples were taken per time point and treatment, each sample consisting of three leaves pooled from one plant. Plant zinc, nickel and cadmium treatments were significant predictors of *Psm* growth at both day 2 and day 5 (ANOVAs; *P*<0.0005). Bonferroni simultaneous comparisons were carried out; means that were not significantly different are marked with the same letter. Values are means ± SE (*n* = 6). The experiment was repeated twice with similar results.

*T. caerulescens* is able to accumulate three metals, zinc, nickel and cadmium, in its shoots. To determine whether nickel and cadmium also conferred increased resistance to infection, bacterial growth was quantified in leaves of *T. caerulescens* plants grown on nutrient solution supplemented with different concentrations of zinc, nickel, or cadmium. For all three metals, increasing metal concentration resulted in reduced bacterial growth. Zinc treatments at concentrations ≥30 µM, and nickel and cadmium treatments at concentrations ≥10 µM, caused significant inhibition of bacterial growth at 2 and 5 days post-inoculation (Figure 3). Accumulation of any of these metals therefore inhibits bacterial growth *in planta* and defends *T. caerulescens* against disease.

Studies of plants that are not normally exposed to high concentrations of metals have shown that metal treatment can induce a range of stress responses, including up-regulation of genes associated with local plant defense responses and systemic acquired resistance (SAR) such as *PR-1* [31]. In order to determine whether some of the protection conferred by high zinc was a consequence of the effect of high zinc concentrations on defense-associated gene expression, we compared the expression of *PR-1* in leaves of *T. caerulescens* plants grown on 0.04 and 300 µM zinc by quantitative real-time PCR. Normalized *PR-1* expression in the two metal treatments was similar to *PR-1* expression in healthy *A. thaliana* leaves (relative expression levels in 0.04 µM, 1.423±0.299; 300 µM Zn, 1.942±0.520; untreated *A. thaliana*, 2.102±1.435), and there was no significant increase in expression in response to increased zinc (Figure S2). This indicates that the inhibitory effect of increasing metal treatments on *Psm* growth is not due to up-regulation of SAR.

### Apoplast extracts from *Thlaspi caerulescens* plants grown on high metal concentrations show decreased ability to support bacterial growth

**Figure 4:**
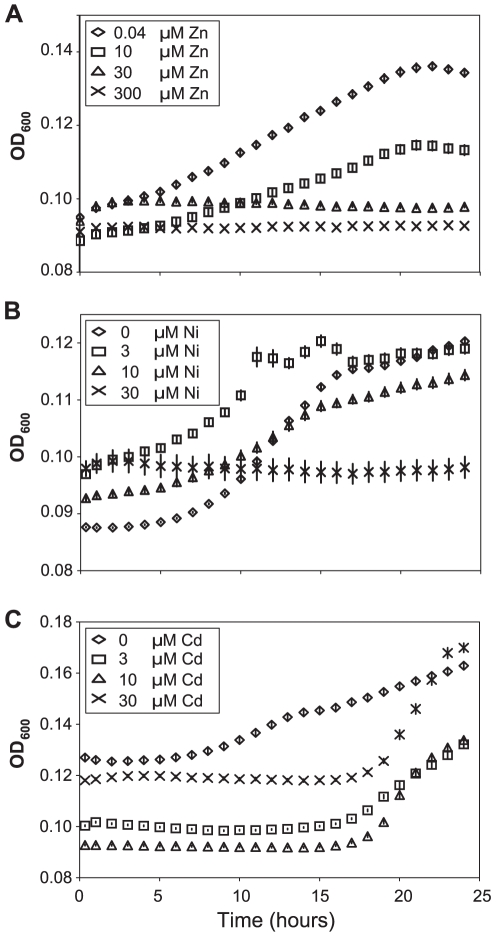
Apoplast extracts from *Thlaspi caerulescens* plants grown in high metal concentrations inhibit bacterial growth. Apoplast extracts obtained from *T. caerulescens* plants treated with the same zinc (**A**), nickel (**B**), or cadmium (**C**) concentrations used in the bacterial colonization assays shown in Figure 3 were used as a growth medium for *P. syringae* pv. maculicola M4. Six samples of 100 µl of apoplast were used for each treatment. Values are means ± SE (*n*  = 6). The experiment was performed three times with similar results, and the results shown are from one experiment representative of the three. Concentrations of all three metals were significant predictors of growth at 18 and 24 hours (ANOVAs: *P*<0.0005 in all cases except *P* = 0.015 for cadmium at 24 h). Bonferroni simultaneous comparisons (α = 1%) show that all zinc and nickel treatments resulted in significantly less growth than the control at 18 h and 24 h, as did all cadmium treatments up to 18 h.

*Psm* infects the apoplastic phase between plant cells. To test whether metal accumulation reduces the suitability of this environment for bacterial growth, *Psm* was cultivated *in vitro* in apoplastic fluid extracted from plants treated with supplementary metals. Apoplast extracts from zinc- and nickel-treated plants supported significantly less bacterial growth than those from plants grown without supplementary metal (Figure 4A and B). Apoplast extracts from plants treated with higher cadmium concentrations also inhibited bacterial growth for up to 18 hours, although this inhibition was eventually released (Figure 4C). As such, it is clear that metal hyperaccumulation by *T. caerulescens* makes the apoplast a more hostile environment for the growth of *Psm*.

### Metal concentrations in *Thlaspi caerulescens* leaves and extracted apoplast are sufficient to explain the high disease resistance of metal-treated plants

**Figure 5:**
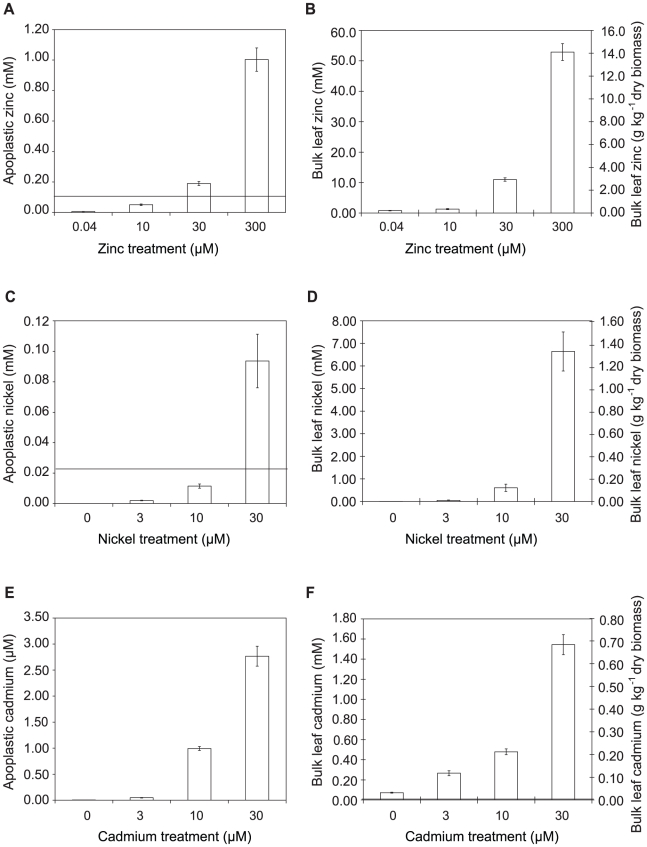
Metal concentrations in zinc-, nickel- and cadmium-treated plants. Data represent average values from three independent sets of plants. Metal content was determined by atomic absorption spectrophotometry. **A, C, E**: Apoplastic concentrations of zinc, nickel and cadmium, respectively. **B, D, F**: Bulk-leaf concentrations of zinc, nickel and cadmium, respectively, expressed both as molar concentration calculated on the basis of leaf fresh biomass, and as mass concentration relative to leaf dry biomass. Values are means ± SE (*n* = 6). Horizontal bars in **A**, **C** and **F** indicate metal concentrations representing the respective IC_50_ values for *P. syringae* pv. maculicola M4 determined experimentally in extracted apoplast (i.e. Zn = 0.12 mM; Ni = 0.025 mM; Cd = 0.01 mM); IC_50_ values measured in LB were higher (Zn = 0.63 mM; Ni = 0.78 mM; Cd = 0.22 mM).

To determine whether hyperaccumulated metal could be responsible for the observed reductions in bacterial growth in leaves, the ability of *Psm* to tolerate each metal was tested in a range of synthetic media and in extracted apoplast (Figure S3) and compared with the metal concentrations of apoplast extracts and whole-leaf tissue samples (Figure 5; Table 1). For all metals tested, concentrations in whole-leaf tissue of *T. caerulescens* plants grown at the two highest treatments were sufficient (i.e. higher than the IC_50_ values for the respective metals) to explain the observed reduction in bacterial growth *in planta*. Apoplastic zinc and nickel concentrations from the highest treatments were also sufficient to explain bacterial growth reduction. Apoplastic cadmium concentrations were considerably lower, reaching around one-third of the IC_50_ concentration for *Psm in vitro* in higher treatments, perhaps explaining the ability of *Psm* eventually to overcome inhibition by cadmium.

**Table 1:** Summary of bacterial growth across treatments in relation to metal concentration and metal-dependent growth inhibition of *P. syringae* pv. maculicola *in vitro*.

Treatment	Growth a		Metal Concentration (mM) b	
	*in planta*	in apoplast	Whole Leaf	Apoplast
0.04 µM Zn	5.78±0.31.	0.036±0.0025 …	0.814±0.281	0.0058±0.002
10 µM Zn	5.44±0.19	0.022±0.0020 **	1.291±0.458*	0.0505±0.010
30 µM Zn	3.77±0.30***	0.004±0.0006***	11.00±0.731*	0.1898±0.025*
300 µM Zn	3.47±0.13 ***	0.002±0.0001***	52.89±4.522*	1.0034±0.063*
0 µM Ni	5.57±0.36	0.028±0.0007	0.004±0.004	0.0001±0.000
3 µM Ni	5.63±0.16	0.020±0.0020**	0.056±0.025*	0.0020±0.000
10 µM Ni	4.45±0.24 ***	0.018±0.0011***	0.607±0.381*	0.0115±0.003*
30 µM Ni	3.63±0.19**	0.000±0.0014***	6.641±2.489*	0.0937±0.031*
0 µM Cd	6.31±0.08	0.024±0.0235	0.073±0.008 *	0.0000±0.000
3 µM Cd	5.63±0.09	0.006±0.0060***	0.226±0.064*	0.0001±0.000
10 µM Cd	4.27±0.03 ***	0.004±0.0035***	0.479±0.036*	0.0010±0.000
30 µM Cd	4.07±0.16***	0.003±0.0032***	1.544±0.1246*	0.0028±0.000

a ‘Growth’ indicates mean log bacterial cells/cm^2^ leaf 5 days after inoculation (*in planta*) or increase in OD over 18 hours *in vitro* in extracted apoplast. Means found to be significantly different from the control mean (Bonferroni simultaneous comparisons) are marked with *(*α* = 5%), **(*α* = 1%), or ***(*α* = 0.1%). In the case of Ni *in planta* data, the 3 µM treatment was used as a control for statistical analysis because of the relatively high variability of the 0 µM treatment.

b Metal concentrations of either bulked whole leaf samples or of the apoplast are shown. * indicates that concentrations were equal to or greater than the IC_50_ values for the respective metals for *Psm* in extracted apoplast.

### *Pseudomonas syringae* pv. maculicola zinc tolerance mutants show differential growth in *Thlaspi caerulescens*

**Figure 6:**
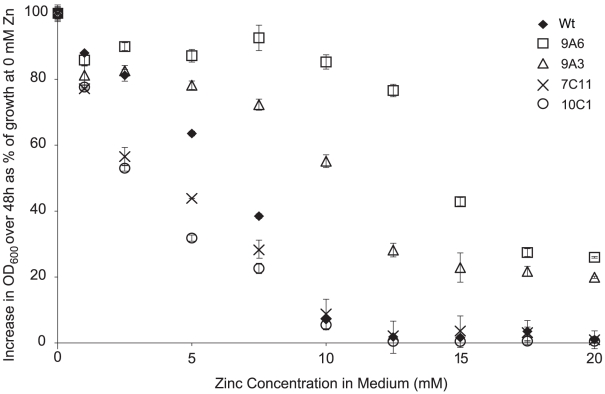
Zinc tolerance of *P. syringae* pv. maculicola M4 and four mutants. Five µl of bacterial suspension at an OD_600_ of 0.2 were inoculated into 200 µl of KB broth supplemented with zinc at 0 to 20 mM. The graph shows the percentage increase in OD_600_ 48 hours after inoculation, relative to the increase in OD_600_ over 48 hours observed for the same strain in the absence of zinc. At least four samples were analysed for each treatment. Values are means ± SE (*n* = 8).

The importance of metal tolerance for bacteria growing in metal-hyperaccumulating *T. caerulescens* was assessed by screening a transposon mutant library of *Psm* to identify mutants with increased or decreased zinc tolerance relative to wild-type *Psm*. The performance of four representative mutants *in planta* was then compared to that of the wild-type strain under four zinc regimes. Two mutants with increased zinc tolerance (9A6 and 9A3) and two with reduced zinc tolerance (10C1 and 7C11) were used (Figure 6). These four mutants grew similarly to wild-type *Psm* in *A. thaliana* (Figure S4) and were able to cause similar symptoms to wild-type *Psm* in *A. thaliana* and pak choi, *Brassica rapa* ssp. *chinensis* (data not shown). Mutant 7C11 showed slightly reduced growth relative to wild-type *Psm* during late log phase and stationary phase in *in vitro* growth assays in KB broth (Figure S5), but the *in vitro* growth kinetics of the other three mutants were not significantly different from wild-type *Psm*.

The disrupted genes were sequenced using an inverse PCR method and their predicted functions are described in Table 2. Interestingly, mutant 7C11 was found to contain an insertion in a TonB-dependent siderophore receptor (PSPTO_2152), which suggests that the slight growth defect observed in KB broth for this strain may be linked to impaired iron acquisition. The other mutation giving rise to reduced zinc tolerance, in mutant 10C1, was located in an NAD-dependent DNA ligase gene (PSPTO_0382); this does not have an obvious role in metal transport or metal tolerance, but is located close to two operons predicted to encode a heavy-metal-sensing two component regulatory system and components of a cobalt-zinc-cadmium (Czc) cation efflux system (PSPTO_0375 – PSPTO_0379) in the genome of *P. syringae* pv. tomato DC3000. The two mutations giving rise to increased zinc tolerance were located in *pslF* (PSPTO_3533; 9A3), a gene within the *psl* operon, involved in exopolysaccharide synthesis and biofilm formation in *Pseudomonas aeruginosa* [32], and in a proline iminopeptidase (*pip*) gene (PSPTO_5164; 9A6). The *pip* gene of *Xanthomonas campestris* pv. campestris has been shown to be induced during plant colonisation and to be essential for pathogenesis on cabbage [33]. However, our results indicate that the *pip* gene of *Psm* is not required for pathogenesis in *A. thaliana*. Pip belongs to a family of metalloproteases and its enzymatic activity may be dependent on a metal cofactor such as zinc or cobalt. In the genome of *P. syringae* pv. tomato DC3000, *pip* is located upstream of, and in a putative operon with, a predicted D-Tyr-tRNAtyr deacylase, which may have a role in protecting cells against D-tyrosine toxicity. In *E. coli*, zinc and D-tyrosine have been shown to have opposite effects on the phosphatase activity of the aromatic amino acid biosynthesis regulator TyrR, which is stimulated by zinc and suppressed by L-tyrosine and D-tyrosine [34].

**Table 2:** *P. syringae* pv. maculicola M4 mutants with altered zinc tolerance.

Mutant ID	IC_50_ (mM zinc) in KB Broth	Top BLAST Hit GenBank ID	Expectation Value	Best Match in *P. syringae* pv. tomato DC3000	Predicted Function
9A3	12	NP_793313.1	3.00E−42	PSPTO_3533	Glycosyl transferase
9A6	10	NP_794895	6.00E−57	PSPTO_5164	Proline iminopeptidase
7C11	3.8	NP_791973	2.00E−65	PSPTO_2152	TonB-dependent siderophore receptor
10C1	2.3	NP_790231.1	7.00E−124	PSPTO_0382	NAD-dependent DNA ligase
Wild-type	7.3	N/A	N/A	N/A	N/A

**Figure 7:**
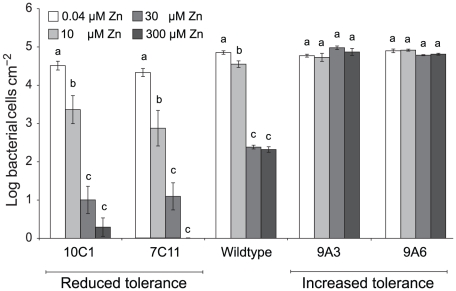
*P. syringae* pv. maculicola M4 mutants with altered zinc tolerance show differential growth in *Thlaspi caerulescens.* *T. caerulescens* plants were treated with 0.04, 10, 30, or 300 µM zinc. Nine leaves of three plants were infiltrated with either *P. syringae* pv. maculicola M4 or one of four zinc-tolerance mutants (9A6, 9A3, 7C11, or 10C1) suspended in 10 mM MgCl_2_ at 10^6^ cfu/ml. Leaves were sampled for bacterial counts at 0 and 5 days after inoculation. Each replicate consisted of three leaves from one plant, giving a total of three replicates per time point and treatment. Values are means ± SE (*n* = 3). The experiment was performed three times with similar results, and the results shown are from one experiment representative of the three. ANOVAs were used to test for a significant effect of plant zinc treatment on the growth of each bacterial strain. No effect was detected for the high tolerance mutant 9A3 (*P* = 0.13). For the other strains, growth was found to be dependent on zinc (9A6: *P* = 0.01; wild-type, 7C11 and 10C1: *P*<0.0005 in each case). Where a significant effect was found, Bonferroni simultaneous comparisons (α = 5%) were carried out. Within each strain, means marked with the same letter were not significantly different.

The ability of the four *Psm* mutants to grow in *T. caerulescens* plants cultivated on 0.04, 10, 30, or 300 µM zinc was found to vary according to the zinc tolerance of the inoculated strain (Figure 7). Thus, while the wild-type showed significant growth reduction with plant zinc treatments of 30 µM or higher, the two mutants with increased zinc tolerance, 9A6 and 9A3, were capable of growth in plants treated with 300 µM zinc. The two mutants with decreased zinc tolerance, 7C11 and 10C1, were unable to grow even in plants treated with 10 µM zinc, at which concentration the wild-type grew well.

Because increased zinc tolerance could be correlated with reduced zinc uptake, we tested the ability of all four mutants to grow at low zinc concentrations. Wild-type *Psm* grew equally well in M9 minimal medium and in M9 supplemented with 1 to 10 µM Zn. Of the four mutants, only 9A6 had an increased zinc requirement relative to wild-type bacteria, showing optimal growth at concentrations ranging from 1 to 5 µM zinc.

### Naturally occurring endophytes of *Thlaspi caerulescens* show high zinc tolerance

**Figure 8:**
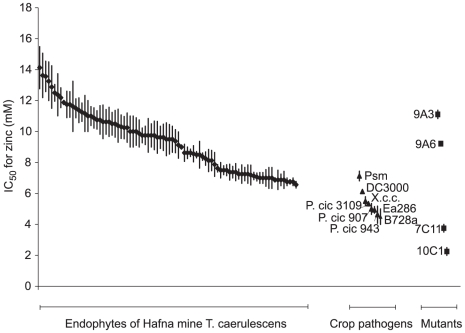
Bacterial endophytes isolated from a natural population of *Thlaspi caerulescens* exhibit high zinc tolerance. To determine IC_50_ values for zinc, 5 µl of bacterial suspension at an OD_600_ of 0.2 were inoculated into 200 µl of KB broth supplemented with zinc at 0 to 20 mM. OD_600_ was measured after incubation, with continuous shaking, at 28°C for 48 hours, and IC_50_ values were calculated from the resulting dose–response curves. Values are means ± SE (*n* = 3). Diamonds  =  Hafna mine endophytes; triangles  =  plant pathogenic bacteria isolated from non-metal-accumulating crop plants; squares  =  mutants of *P. syringae* pv. maculicola generated in the present work. Abbreviations: DC3000, *Pseudomonas syringae* pv. tomato DC3000; *X.c.c.*, *Xanthomonas campestris* pv. campestris 8004; *P. cic* 3109, *Pseudomonas cichorii* NCPPB3109; *P. cic* 907, *P. cichorii* NCPPB907; *P. cic* 943, *P. cichorii* NCPPB943; Ea286, *Erwinia amylovora* Ea286; B728a, *P. syringae* pv. syringae B728a.

To test further the hypothesis that high metal tolerance is a prerequisite for bacterial growth in *T. caerulescens*, endophytic bacteria were collected from leaves of a natural population of *T. caerulescens* plants at Hafna Mine, a former lead–zinc mine in North Wales, UK [35], and their zinc tolerance assessed. The mean zinc content of the leaves of these plants was 16.2±1.39 g Zn per kg leaf dry mass (± s.e.m., *n* = 23 leaves), slightly greater (*p*<0.05) than that of plants treated with the highest concentration of zinc (300 µM) used in the laboratory experiment (13.7±0.71 g Zn per kg dry mass (± s.e.m., *n* = 18 leaves)). The average IC_50_ for zinc of these naturally occurring endophytes was 9.4 mM (*n* = 86) in KB medium. In contrast, a set of plant pathogenic bacteria isolated from non-hyperaccumulating plants were found to have a significantly (*p*<0.001) lower mean IC_50_ for zinc of 5.4 mM in KB (*n* = 7; Figure 8). When the zinc IC_50_ values for individual strains isolated from the Hafna mine plants were compared to the zinc IC_50_ of *Psm*, the most zinc tolerant of the plant pathogens used in this study, 65% were found to have a significantly higher zinc tolerance (*p*≤0.05), while only a single strain out of 85 had a significantly lower zinc tolerance. Therefore, in the field, as in the laboratory, only metal-tolerant bacteria can colonize *T. caerulescens*.

## Discussion

In this work, we have developed a model system to study the elemental defense hypothesis for plant metal hyperaccumulation using the bacterial pathogen *Pseudomonas syringae* pv. maculicola M4. We have shown that *Psm* displays T3SS-dependent pathogenesis in *Thlaspi caerulescens* plants grown in low metal concentrations and that metal hyperaccumulation by *T. caerulescens* at higher metal concentrations provides an effective defense against *Psm*. Further, we have demonstrated that zinc tolerance is essential for bacterial colonization of zinc-hyperaccumulating plants. Thus, our work is consistent with the hypothesis that hyperaccumulation benefits plants by increasing their resistance to pathogens.

Although studies have found that zinc, nickel and cadmium are mainly stored in the leaf cell vacuoles of *Thlaspi* species, particularly in epidermal cells [27], [28], [36], [37], [38], metals are transported into the leaf through the extracellular spaces of the apoplast by means of the transpiration stream. By measuring metal concentrations in the apoplastic fluid, we were able to provide an approximation of the conditions experienced by invading pathogens in the leaves of *T. caerulescens*. Comparison of the metal concentrations *in planta* with the IC_50_ values measured for *Psm in vitro* in extracted apoplastic fluid allowed us to demonstrate that direct inhibition of bacterial growth by hyperaccumulated zinc or nickel is a realistic possibility. *T. caerulescens*, when grown on at least 10 µM Zn or 30 µM Ni, accumulated sufficient metal in apoplastic fluid to provide an elemental defense against *Psm*. This result is particularly striking considering that, when grown at 10 µM Zn, *T. caerulescens* accumulated only an average of 0.3 g Zn per kg dry mass, while the threshold for designation as a zinc hyperaccumulating plant is 10 000 mg per kg [1]. This indicates that *T. caerulescens* could be protected against pathogens by zinc accumulation even when growing on relatively low-zinc soils. However, although *T. caerulescens* accumulates sufficiently high metal concentrations to account for its metal-dependent resistance to *Psm*, we cannot rule out the possibility that additional metal-dependent factors act in conjunction with metals to limit the growth of *Psm* in *T. caerulescens*. Accumulation of metals requires mechanisms by which the plant may tolerate elevated intracellular metal concentrations, which have not been fully elucidated, but which have been shown to involve redox-related compounds such as glutathione [39], enzymes such as superoxide dismutase [40], and metal-binding ligands such as organic acids and amino acids [27], [41], as well as proteins (e.g. metallothioneins [42]). Such changes may affect the quality of the plant environment for growth of *Psm* or zinc tolerance in *Psm*, without having any expressly defensive function.

One case in which additional factors seem likely to contribute to metal-dependent defenses is in plants treated with cadmium. Cadmium was able to defend *T. caerulescens* against *Psm in planta* when plants were treated with ≥10 µM cadmium. Bacterial growth was also inhibited for around 18 hours in apoplastic fluid extracted from these plants. After this time, inhibition was released, possibly as a result of changes in bacterial gene expression or in the composition of the apoplast during this time *in vitro*, which may include alterations in cadmium bioavailability. However, the cadmium concentrations detected in apoplast extracts from cadmium-treated plants were lower than those required for inhibition of bacterial growth in apoplast extracts from plants grown in the absence of cadmium, so it is possible that cadmium concentrations are correlated with another defensive factor which may be unstable (such as ROS) or volatile.

To investigate further the possibility of a true elemental defense, we tested the importance of zinc tolerance for bacterial growth *in planta* in zinc-treated *T. caerulescens*. The results obtained using mutants of *Psm* provide the clearest evidence to date of a direct role for zinc as an elemental defense against pathogens in *T. caerulescens*. Mutants with reduced zinc tolerance were unable to multiply in plants grown at 10 µM zinc, in leaves of which their wild-type counterpart was successful; conversely, mutants with increased zinc tolerance grew well in leaves of plants grown at 30 µM or even 300 µM zinc, in which the wild-type could not survive. This clear link between bacterial zinc tolerance and ability to colonize plants hyperaccumulating zinc provides strong support for the concept of an elemental defense by zinc in *T. caerulescens*.

Finally, we have compared the zinc tolerance of strains used in this work with that of bacteria isolated from the leaves of *T. caerulescens* plants growing under natural conditions in a zinc-polluted field site. We have shown that bacteria naturally colonizing these plants in the field had a range of zinc tolerances much higher than those of plant pathogenic strains isolated from non-accumulating crop plants, providing evidence for local adaptation of these endophytes to their environment [43], [44], [45]. This is in agreement with previous studies demonstrating that endophytic bacteria isolated from nickel hyperaccumulators exhibit high nickel tolerance [46], [47]. Plant pathogens that have not been subject to this selection may find it difficult to grow and cause disease in *T. caerulescens*.

Metal-dependent resistance in *T. caerulescens* may be particularly effective against airborne, foliar pathogens such as *P. syringae* and powdery mildew, which may be deposited onto the surface of *T. caerulescens* leaves by rain and wind having previously colonized non-accumulating or metal-excluding plants, with no prior selection for metal tolerance. Thus, hyperaccumulated metals may be functionally equivalent to the diverse array of anti-microbial secondary metabolites used by plants to provide protection against infection. Only pathogens that are able to tolerate or inhibit the chemical defenses present in a specific plant species or genotype can grow in plant tissues. Similarly, in the case of metal-hyperaccumulation, only a small number of organisms – those possessing high metal tolerance – are able to grow in these plants. When the plants are deprived of this form of defense by cultivation on a low-metal growth medium, they may become vulnerable to a wider range of pathogens, explaining the spontaneous outbreaks of mildew infection observed on such plants (Figure 1).

When considering the role of metals in protecting plants against infection in a natural setting it is important to note that metal concentrations in the leaves of hyperaccumulating plants are typically orders of magnitude higher than metal concentrations in the environment, as illustrated in Figure 5. Therefore, even though the environment surrounding these plants may favour the growth of moderately metal tolerant microorganisms, many of these organisms may have insufficient metal tolerance to be able to grow in the tissues of hyperaccumulating plants. In addition, the observation that T3SS mutants of *Psm* were unable to infect *T. caerulescens* plants grown on low metal concentrations indicates that the plants possess additional defense mechanisms that act in conjunction with metal hyperaccumulation to protect plants, but which can be suppressed by the action of T3SS effectors. Thus, successful pathogens of *T. caerulescens* must not only be adapted for growth in a metal-rich environment, but must also possess at least some of the pathogenicity mechanisms known to be required for infection of non-accumulating plants such as *Arabidopsis thaliana*.

We have demonstrated that hyperaccumulation of any of three metals, zinc, nickel, or cadmium, by *T. caerulescens* provides the plant with an elemental defense against the hemibiotrophic pathogen *Psm*. The validity of the defense hypothesis has been challenged by some studies in which no evidence was found that metal hyperaccumulation defended *T. caerulescens* from herbivory in the field [13], [14]. Our work, however, suggests that the defensive effect of metal hyperaccumulation against pathogens remains relevant in field conditions, with only metal-tolerant bacteria found growing naturally in the leaves of *T. caerulescens.* Further, we have shown that metal concentrations in the leaves are sufficient to account for this defensive effect without invoking any other factors. For all of the metals hyperaccumulated by *T. caerulescens*, we have shown that growth is also inhibited in apoplastic fluid, and that both zinc and nickel are found in this specific compartment at concentrations sufficient to account for the defensive effect. Moreover, we have demonstrated that the zinc tolerance of *Psm* mutants is correlated with their ability to colonize zinc-hyperaccumulating *T. caerulescens* plants. This result is mirrored by our findings concerning the zinc tolerance of natural endophytes of *T. caerulescens* from a zinc-rich field site. We therefore believe that metal hyperaccumulation by *T. caerulescens* can provide an effective form of defense against a wide range of pathogens.

## Materials and Methods

### Plant material

Seeds of *Thlaspi caerulescens* J. & C. Presl from Prayon, Belgium (provided by A.J.M. Baker and C. Lefèbvre) were cultured hydroponically on modified 0.1-strength Hoagland solution [20] in a glasshouse. The Prayon population of *T. caerulescens* was chosen as it is a widely studied, well characterized population showing typical hyperaccumulation behavior and producing relatively large amounts of biomass under the growth conditions described [20], [22], [24], [25]. Natural radiation was supplemented by sodium-vapor lamps for 14 hours per day. Night temperature was maintained at 14°C and day temperature at a minimum of 24°C. Two-week-old plants were transferred to modified 0.1-strength Hoagland solution containing 0.04, 10, 30, or 300 µM ZnSO_4_, or 0, 3, 10, or 30 µM NiSO_4_ or CdSO_4_. The lowest zinc concentration used was 0.04 µM, rather than 0 µM for Ni and Cd. This is because zinc is an essential micronutrient without which the plants do not survive. The Hoagland solution used for all Ni and Cd assays contained 10 µM Zn for this reason; at 0.04 µM Zn, signs of zinc deficiency become apparent. *T. caerulescens* plants were grown on these metal treatments for a further 8 weeks, the nutrient solution being exchanged fortnightly for the first 6 weeks and weekly for the final 2 weeks. *Arabidopsis thaliana* (L.) Heynh. (Col-0) were sown on peat-based compost and grown in a glasshouse under the same conditions for 6 weeks.

### Bacterial growth conditions, media and antibiotics

Bacterial strains used in pathogenicity assays are listed in Table S1. *Psm* ES4326, *Psm hrpN^−^* and *Psm hrpS^−^* were provided by K. Schreiber and D. Desveaux. *Psm hrpN^−^* and *Psm hrpS^−^* were isolated from a transposon library of *Psm* ES4326 constructed using a kanamycin-resistant derivative of mini-Tn5 ([48]; D. Desveaux, personal communication). All bacterial strains were streaked onto Luria–Bertani (LB) agar [49] from stocks kept in glycerol at −80°C and incubated at 28°C (37°C for *Escherichia coli*) for 24 to 48 hours prior to use. Single colonies were transferred to LB broth and incubated at 28°C with shaking for most applications. King's B (KB) agar [50], supplemented with CFC (Cetrimide-Fucidin-Cephalosporin; Oxoid) at half the manufacturer's recommended concentration, was used to selectively culture bacteria isolated from plant tissues for *in planta* growth studies. LB and KB broths were used in metal-tolerance experiments. For zinc-requirement assays, the minimal medium M9 [49] was used.

### Bacterial growth *in vitro*

To compare the *in vitro* growth of wild-type and mutant strains of *Psm*, bacteria were grown overnight on LB agar and resuspended in KB broth to give an OD_600_ of 0.1. One hundred microliters of this suspension were added to a further 100 µl of media in each of 16 wells of a 96-well microplate. Sixteen wells were inoculated with media alone as a media control. OD was measured every 20 min for the next 48 hours using an Infinite M200 plate reader (Tecan Group Ltd., Männedorf, Switzerland).

### Pathogenicity assays and bacterial growth *in planta*

For assays to examine the ability of bacteria to cause disease symptoms in *T. caerulescens*, bacteria were grown overnight on LB agar and re-suspended in sterile 10 mM MgCl_2_ at an optical density at 600 nm (OD_600_) of 0.35. Bacterial suspensions were infiltrated into at least three fully expanded leaves from 6-week-old *T. caerulescens* plants grown on nutrient solution (10 µM zinc) or 6-week-old *A. thaliana* using a blunt 1 ml syringe. Symptoms resulting from bacterial inoculation were monitored over 7 days. For growth assays, bacteria were resuspended in sterile 10 mM MgCl_2_ at an OD_600_ of 0.2. This suspension was diluted 100-fold to give a suspension of approximately 10^6^ cfu/ml and infiltrated into fully expanded *T. caerulescens* or *A. thaliana* leaves through the abaxial surface using a blunt 1 ml syringe. Nine leaves on each of six plants were inoculated within each metal treatment. Leaf discs of 10-mm diameter were taken from three of the inoculated leaves immediately, and from three further leaves at 2 and 5 days after inoculation. Leaf discs were homogenized in 10 mM MgCl_2_ and the resulting suspension spread onto agar plates with a minimum of three technical replicates used for each sample. After incubation at 28°C for 48 hours, the number of bacterial colonies was counted and used to estimate the number of bacterial cells per unit area of leaf.

### Apoplast extracts

Apoplastic fluid was extracted from *T. caerulescens* and *A. thaliana* leaves by a modification of the method described by Rico and Preston [51], using vacuum infiltration of the intercellular spaces with distilled water followed by centrifugation to extract apoplastic fluid. This fluid was centrifuged for a further 10 minutes at 4°C, filter-sterilized, and stored at −80°C. The degree of apoplast dilution was estimated as described by Rico and Preston [51]. For bacterial growth experiments, apoplast extract was freeze-dried and resuspended in an appropriate volume of distilled water to return it to its estimated concentration *in planta*.

### Bacterial growth in apoplast extracts

Apoplast extracts from *T. caerulescens* plants were used as a substrate for the growth of *Psm in vitro.* Six 100 µl samples of apoplast extract from each metal treatment were aliquoted into a 96-well microwell plate and inoculated with 5 µl of *Psm* suspended in 10 mM MgCl_2_ to give a final OD_600_ of 0.05. The plate was incubated at 28°C with shaking and the OD_600_ measured at 20-minute intervals for 24 hours using the plate reader.

### Metal content of whole-leaf and apoplast extracts of *Thlaspi caerulescens*

For determination of whole-leaf metal concentrations, fresh leaf material was oven-dried at 80°C for 48 hours. Subsamples of 50 mg of dried leaf material were digested in 3 ml of concentrated (69%, v/v) nitric acid for 16 hours in glass vials. Samples were then diluted 10-fold with ultrapure water and filtered using Whatman grade 3 filter paper. Metal contents of samples were measured in an air–acetylene flame by atomic absorption spectrophotometry using a double-beam optical system with deuterium arc background correction (AAnalyst 100; Perkin-Elmer, UK). Samples were further diluted as necessary to fall within the linear range of calibration curves prepared using appropriate standard solutions and reagent blanks. The accuracy of the calibration curves was validated using Certified Reference Material LGC7162 (strawberry leaves; LGC Standards, Teddington, UK). Three technical replicates were analysed for each measurement, and two samples from each of three plants were analysed for each metal treatment. Measurements of the fresh and dry biomass of 24 individual *T. caerulescens* plants were used to provide an average ratio between fresh and dry biomass, which allowed the metal content to be expressed as an approximate molar concentration in the fresh tissue. Apoplast samples were diluted appropriately with ultrapure water and measured without further treatment.

### Metal-tolerance assays

The metal tolerance of *Psm in vitro* was tested in LB. A 5 µl aliquot of an overnight liquid culture was added to 200 µl of media supplemented with zinc, nickel, or cadmium at a range of concentrations. OD_600_ was read using the plate reader. Metal tolerance was also tested in apoplast extracted from *T. caerulescens* plants grown in 0.1-strength Hoagland solution (10 µM zinc). For these experiments, 2 µl of an overnight culture of *P. syringae* pv. maculicola M4 was added to 75 µl of apoplast extract or apoplast extract supplemented with metal.

### Transposon mutagenesis of *Pseudomonas syringae* pv. maculicola M4

Transposon mutagenesis was carried out using the transposon miniTn5*::gfp::lux* cloned into pGP704 (generous gift of Phil Hill). The transposon was introduced into *Psm* by triparental mating using the helper plasmid pRK2013 [52]. The resultant bacterial mixture was spread onto LB agar containing 50 µg/ml kanamycin and 50 µg/ml rifampicin. Resulting colonies were inoculated into 150 µl of KB broth in 96-well plates and incubated at 28°C overnight. Thirty microliters of 50% (v/v) glycerol was then added to each well and plates were stored at −80°C as a mutant library.

### Screening the *Pseudomonas syringae* pv. maculicola mutant library for zinc tolerance mutants

Mutants were screened in a four-stage process. In round one, mutants were grown in KB in 96-well plates in which a wild-type was also included. A 5 µl aliquot of overnight culture was transferred to 200 µl of KB in a black, clear-bottomed 96-well plate and incubated at 28°C in the plate reader. Plates were maintained at 28°C with continuous shaking and the OD_600_ and luminescence from each well was read at hourly intervals. After 3.5 hours, cultures were supplemented with 5 µl of 0.5 mM ZnSO_4_. OD_600_ and luminescence were read immediately and then at intervals of 3 to 9 hours for the next 48 hours. Changes in luminescence after the addition of zinc were recorded and growth was compared to wild-type.

A total of 866 strains were selected with markedly increased or decreased tolerance to zinc shock. Each of these mutants was transferred to 200 µl KB in two separate wells of a 96-well plate and growth was monitored with and without 0.5 mM zinc over 48 hours. Of these, 134 mutants whose tolerance differed notably from the wild-type were selected and further screened for growth in 200 µl KB in 96-well plates with zinc concentrations from 0 to 3.5 mM for 48 hours. IC_50_ values were then calculated and compared to wild-type. These results were validated in a second experiment for ten mutants that showed the largest consistent changes in zinc tolerance compared to wild-type. These ten mutants were then tested for their ability to cause symptoms in *T. caerulescens*, *A. thaliana* and pak choi (*Brassica rapa* spp. *chinensis*).

### Inverse-PCR based sequencing of transposon mutants

Genomic DNA was extracted using a DNeasy Blood and Tissue kit (Qiagen) according to the manufacturer's protocol for Gram negative bacteria. Eight microliters of the subsequent DNA suspension were digested with 1 µl of either the restriction endonuclease *Sph*I or *Nar*I (New England BioLabs) and 1 µl of the appropriate 10× buffer according to the manufacturer's specifications. *Nar*I cleaves the transposon DNA near the 3′ end, while *Sph*I cleaves it towards the 5′ end; both also cleave the *Psm* genome frequently. Digested DNA was ethanol-precipitated, resuspended in 8 µl of ultrapure water, and self-ligated overnight at 14°C using 1 µl of T4 ligase and 1 µl of 10× buffer (New England BioLabs). One µl of circularized DNA was then used as a template for inverse PCR. Primers designed to the ends of the short fragments of transposon resulting from *Nar*I (AACAATCTAGCGAGGGCTTGGTAAGGTGATCC and CTTGCAGTGGGCTTACATGACGATAGCTAGAC) or *Sph*I (GGAACGCCGCAGGAATG and CAGCAGCTGTTACAAACTCAAGAAG) digestion, and facing outwards, were used to amplify the flanking DNA. This was then sequenced using a primer to the end of the transposon (CGGTTTACAAGCTAAAGCTTGC for *Nar*I-digested DNA and either CTTCTTTAAAATCAATACC or TTCCAGTAGTGCAAATAA for *Sph*I-digested DNA).

### Assessment of zinc tolerance of naturally occurring endophytes of *Thlaspi caerulescens* from a zinc-rich site

Leaves of *T. caerulescens* were collected in the field at Hafna mine (Snowdonia, North Wales, UK: 53°07′N, 3°49′W). They were then returned to the laboratory and immediately surface-sterilized by immersion in 10% (w/v) sodium hypochlorite for 3 minutes, followed by immersion in 100% ethanol for 3 minutes. Sterile leaves were rinsed in ultrapure water and macerated in 1 ml of 10 mM MgCl_2_ solution. The resulting suspension was plated onto KB-CFC agar. Plates were incubated at 28°C for 48 hours. Resultant colonies were transferred to 150 µl of KB broth in 96-well plates. After 48 hours of growth at 28°C, 30 µl of 50% (v/v) glycerol was added to each well and the plates stored at −80°C. For zinc tolerance experiments, bacteria were grown in 200 µl KB for 48 hours and replicated into 200 µl KB supplemented with 0, 1, 2.5, 5, 7.5, 10, 12.5, 15, or 20 µM ZnSO_4_. Bacterial growth at each of these zinc concentrations was determined by measuring OD_600_ at 0 and 48 hours using the plate reader. Bacterial growth at 48 hours was then plotted against zinc concentration, from which the concentrations giving half-maximal inhibition (IC_50_) were estimated.

### Quantitative RT-PCR of *PR-1* gene expression

RNA for qRT-PCR analysis was isolated from leaves of 10-week-old *T. caerulescens* grown on either 0.04 or 300 µM zinc and from leaves of 6-week-old, non flowering *A. thaliana*. *A. thaliana* leaves inoculated with *Psm* at 10^6^ cfu/ml and incubated for 24 hours under standard plant growth conditions were used as a positive control for *PR-1* expression. Leaves were snap frozen in liquid nitrogen and RNA was extracted using the RNeasy kit (Qiagen) according to the manufacturer's instructions. After elution, RNA was precipitated in 100 µl of 8 M LiCl overnight. After two washes in 70% (v/v) ethanol, pellets were resuspended in 30 µl ultrapure water. RNA concentration was measured using a Nanodrop-1000 spectrophotometer (Thermo Scientific) and integrity was checked by electrophoresis on an ethidium bromide gel. cDNA was prepared from 1 µg RNA using the Bioline cDNA synthesis kit with oligo dT primer according to the supplier's instructions. qRT-PCR was performed using SYBR green PCR master mix (Applied Biosystems) in a 7300 Realtime PCR machine (Applied Biosystems) and analysed by calibration to a standard curve of gene expression created from pooled cDNA from all samples under test, using the 7300 SDS system software v1.3.1 (Applied Biosystems). Four control genes were analysed in the same way, and *PR-1* gene expression normalized to the geometric mean of the expression of these genes [53]. Gene-specific primers used are listed in Table 3.

**Table 3:** Primers used in qRT-PCR.

Gene product	Forward Primer Sequence	Reverse Primer Sequence	Accession number^a^
PR-1	ACAACTACGCTGCGACGT	TCACTTTGGCACATCCGAGTC	NM_127557
EF1-α	TGAGCACGCTCTTCTTGCTTTCA	GGTGGTGGCATCCATCTTGTTACA	X16430.1
β-tubulin	CACCAGACATAGTAGCAGAAATCAAGT	AAACTCACTACCCCCAGCTTTG	M84702.1
Ornithine transcarbamoylase	TGAAGGGACAAAGGTTGTGTATGTT	CGCAGACAAAGTGGAATGGA	AJ002524.1
UBQ10 (ubiquitin)	AAAGCTCCGACACCATTGAC	CTTATTCATCAGGGATTATACAAGG	NM_178968.4

a GenBank accession numbers for *Arabidopsis thaliana* gene sequences used for primer design (www.ncbi.nlm.nih.gov).

### Accession numbers

The GenBank (http://www.ncbi.nlm.nih.gov) accession numbers for the genes and gene products discussed in this paper are: PSPTO_0382 (NP_790231.1); PSPTO_2153 (NP_791973); PSPTO_3533 (NP_793313.1); PSPTO_5164 (NP_794895).

### Acknowledgments

We thank Prof. A.J.M. Baker for providing seeds of *Thlaspi caerulescens*; Prof. J.L. Dangl for providing *Pseudomonas syringae* pv. *maculicola* M4, Dr. K. Schreiber and Dr. D. Desveaux for providing *hrp* mutants of *Pseudomonas syringae* pv. maculicola ES4326, Dr. P. Hill for proving the transposon used for mutagenesis, and Prof. J.A. Langdale, Prof. N.P. Harberd and Dr. A. Buckling for helpful discussions.

## Supporting Information

Figure S1 *In vitro* growth of wild-type *Pseudomonas syringae* pv. maculicola ES4326 and *hrcN^-^* and *hrpS^-^* mutants. Strains were inoculated into KB broth to give an OD at 600 nm of 0.1, and 100 µl of this suspension was added to a further 100 µl of KB in a 96 well microplate. OD_600_ was monitored over 24 h. Values are means ± SE (*n* = 16).
(0.71 MB EPS)

Figure S2 Expression of *PR-1* in *Thlaspi caerulescens* in response to zinc. Quantitative real-time PCR was used to determine the expression of *PR-1* in *T. caerulescens* grown on 0.04 or 300 µM zinc, and in *A. thaliana*, either after no treatment or 24 hours after inoculation with *Psm* at 10^6^ cfu/ml. Expression was normalised to the geometric mean of the expression of four control genes: ubiquitin, EF-1α, β-tubulin and ornithine transcarbamylase. Within each species, *t*-tests were used to compare the two results obtained. These showed that there was no significant difference between *PR-1* expression in *T. caerulescens* in the two zinc treatments (*P* = 0.2), while inoculation of *A. thaliana* with *Psm* resulted in a significant increase in *PR-1* expression (*P* = 0.005). Values are means ± SE (*n* = 12).
(0.68 MB EPS)

Figure S3 Effect of increasing zinc, nickel and cadmium concentrations on growth of *Pseudomonas syringae* pv. maculicola M4 *in vitro*. Five µl of bacterial suspension at an OD_600_ of 0.2 were inoculated into 200 µl of LB supplemented with varying concentrations of metal. Graphs show the OD_600_ 24 hours after inoculation. A minimum of eight replicates were analysed for each treatment. Values are means ± SE (*n*≥8).
(0.77 MB EPS)

Figure S4 Growth of zinc tolerance mutants of *Pseudomonas syringae* pv. maculicola M4 in *Arabidopsis thaliana*. Six leaves of three plants were infiltrated with either *Psm* or one of four zinc tolerance mutants of *Psm* (9A6, 9A3, 7C11 and 10C1) suspended in 10 mM MgCl_2_ at 10^6^ cfu/ml. Leaves were sampled at 0 and 5 days after inoculation. Each replicate consisted of three leaves from one plant, giving a total of three replicates per time point and treatment. Values are means ± SE (*n* = 5). The experiment was carried out twice with similar results. An ANOVA was used to test for an effect of strain upon growth. This was significant (*P* = 0.033), but Bonferroni simultaneous comparisons showed this significance to be due to a difference between the growth of mutants 9A6 and 9A3; no mutant grew to a level significantly different from that of *Psm* (*P* = 1.000, 1.000, 0.612 and 1.000 for 9A6, 9A3, 7C11 and 10C1, respectively).
(0.47 MB EPS)

Figure S5 Growth of zinc tolerance mutants of *Pseudomonas syringae* pv. maculicola M4 *in vitro*. Strains were added to KB broth to give an OD_600_ of 0.1, and 100 µl of this suspension was added to a further 100 µl of KB in a 96 well microplate. OD_600_ was monitored over 24 h. Values are means ± SE (*n* = 16).
(0.68 MB EPS)

Table S1 Bacteria that caused necrotic symptoms in *Thlaspi caerulescens*.
(0.05 MB DOC)
