# Supplementary material for: Metal Hyperaccumulation Armors Plants against Disease
Source: PLoS Pathog. 2010 Sep 9;6(9):e1001093. doi: 10.1371/journal.ppat.1001093 (PMC2936542; doi:10.1371/journal.ppat.1001093)
Supplement: Table S1 — Bacteria that caused necrotic symptoms in Thlaspi caerulescens. (0.05 MB DOC) [file ppat.1001093.s006.doc]

**Supporting Information**

***Table S1****.* ***Bacteria that caused necrotic symptoms in Thlaspi caerulescens.***

| **Strain^1^** | **Host** | **Reference/Source** | **Degree of necrosis**  **(0 = no necrosis, 3 = severe necrosis)**  **Time after inoculation (days)** | | | | | |
| --- | --- | --- | --- | --- | --- | --- | --- | --- |
|  |  |  | **1** | **2** | **3** | **4** | **5** | **6** |
| *Erwinia amylovora* Ea286 | Pear (*Pyrus communis*) | E. Moltmann | 0 | 0 | 0 | 1 | 1 | 2 |
| *Xanthomonas campestris* pv. *campestris* 8004 | Cauliflower (*Brassica oleracea*) | [1] | 0 | 0 | 0 | 0 | 1 | 2 |
| *Pseudomonas cichorii* NCPPB943 | Endive (*Cichorium endiva*) | W. Kotte, NCPPB^2^ | 0 | 1 | 2 | 2 | 2 | 2 |
| *Pseudomonas cichorii* NCPPB3109 | Coffee (*Coffea arabica*) | C. F. Robbs, NCPPB | 0 | 1 | 1 | 1 | 2 | 2 |
| *Pseudomonas cichorii* NCPPB907 | *Chrysanthemum* sp. | L. A. McFadden, NCPPB | 0 | 1 | 1 | 1 | 1 | 1 |
| *Pseudomonas syringae* pv. tomato DC3000 | Tomato (*Solanum lycopersicum*) | [2] | 0 | 1 | 2 | 2 | 2 | 3 |
| *Pseudomonas syringae* pv. syringae B728a | Bean (*Phaseolus* sp.) | [3] | 0 | 0 | 0 | 1 | 2 | 3 |
| *Pseudomonas syringae* pv. maculicola M4 | Radish (*Raphanus sativus*) | [4] | 0 | 1 | 2 | 3 | 3 | 3 |

^1^ Eight tested bacteria failed to cause symptoms in *T. caerulescens.* They were: *Xanthomonas campestris* pv. campestris NCPPB528 (ATCC33913 [5]), *Xanthomonas campestris* pv. vesicatoria 85-10 [6], *Dickeya dadantii* EC16 (A. Chatterjee), *Pseudomonas viridiflava* PC006 [7], *Pseudomonas marginalis* CTA23 [7], *Pseudomonas* *syringae* pv. syringae 61 (M. Sasser), *Pseudomonas syringae* pv. phaseolicola 1448A [8] and *Pseudomonas aeruginosa* PA14 [9].

^2^ National Collection of Plant Pathogenic Bacteria (NCPBB).

**References**

1. Qian W et al. (2005) Comparative and functional genomic analyses of the pathogenicity of phytopathogen *Xanthomonas campestris* pv. campestris. Genome Res 15: 757–767.

## 2. Buell R et al. (2002) The complete genome sequence of the *Arabidopsis* and tomato pathogen *Pseudomonas syringae* pv. tomato DC3000. Proc Natl Acad Sci USA 100: 10181–10186.

3. Feil H et al. (2005) Comparison of the complete genome sequences of *Pseudomonas syringae* pv. syringae B728a and pv. tomato DC3000. Proc Natl Acad Sci USA 102:11064–11069.

4. Debener T, Lehnackers H, Arnold M, Dangl JL (1991) Identification and molecular mapping of a single *Arabidopsis thaliana* locus determining resistance to a phytopathogenic *Pseudomonas syringae* isolate. Plant J 1: 289–302.

## 5. da Silva AC et al. (2002) Comparison of the genomes of two *Xanthomonas* pathogens with differing host specificities. Nature 417: 459–463.

## 6. Thieme F et al. (2005) Insights into genome plasticity and pathogenicity of the plant pathogenic bacterium *Xanthomonas* *campestris* pv. vesicatoria revealed by the complete genome sequence. J Bacteriol 187: 7254–7266.

## 7. Godfrey SAC, Marshall JW (2002) Identification of cold-tolerant ***Pseudomonas viridiflava*** and ***P. marginalis*** causing severe carrot postharvest bacterial soft rot during refrigerated export from New Zealand. Plant Pathol 51: 155–162.

## 8. Joardar V et al. (2005) Whole-genome sequence analysis of *Pseudomonas syringae* pv. phaseolicola 1448A reveals divergence among pathovars in genes involved in virulence and transposition. J Bacteriol 187: 6488–6498.

# 9. Lee DG et al. (2006) Genomic analysis reveals that **Pseudomonas aeruginosa** virulence is combinatorial. Genome Biol 7: R90.
